# Accessing new avenues of photonic bandgaps using two-dimensional non-Moiré geometries

**DOI:** 10.1038/s41598-023-44385-z

**Published:** 2023-10-10

**Authors:** R. Rachel Darthy, C. Venkateswaran, V. Subramanian, Zhengbiao Ouyang, N. Yogesh

**Affiliations:** 1 Department of Nuclear Physics, School of Physical Sciences, University of Madras, Chennai, 600025 India; 2 Microwave Laboratory, Department of Physics, Indian Institute of Technology Madras, Chennai, 600036 India; 3 Terahertz Technical Research Center, College of Physics and Optoelectronic Engineering, Shenzhen University, Shenzhen, 518060 China; 4 Department of Physics, National Institute of Technology Calicut, Kozhikode, 673601 Kerala India

**Keywords:** Optics and photonics, Photonic crystals

## Abstract

Photonic crystals (PhC) formed by 2-D non-Moiré geometries are realized in this work. Non-Moiré (NM) tiles are the contours of trigonometric functions that generate exciting shapes and geometries**.** Photonic bandstructure calculations reveal that 2-D NM geometries exhibit new avenues of photonic bandgaps compared to the regular circular rod-based PhCs. The band structures are anisotropic and show, intriguing orientation-dependent partial bandgaps. A few of the orientation-dependent frequency selective properties of the realized NM geometry-based PhCs are demonstrated using full-wave electromagnetic simulations. The proposed geometries are practically realizable, and in this work, we experimentally demonstrate the fabrication process using the 3-D printing technique for microwave frequencies.

## Introduction

Photonic crystals (PhCs) have attained central attention ever since the concepts of the bandgap for electromagnetic (EM) frequencies by Yablonovitch^1^ and light localization by the artificial periodic structures demonstrated by John in 1987^2^. PhCs are the periodic arrangement of dielectric constituents in one-, two- and three-dimensions that offer unique control over EM radiation in the form of electromagnetic bandgap (EBG) (i.e., a range of EM frequencies are not allowed inside PhCs), also called photonic bandgap (PBG) and anomalous dispersion (like self-collimation, negative refraction, and super prism) characteristics^3,4^. The PBG region in PhCs plays a vital role in controlling and confining the light with point and line defects in PhCs^5^. Also, there are widespread applications in optoelectronics, optical communications, topological photonics, and all-optical integrated circuits, such as optical waveguides, multiplexers, and couplers^6–12^. Tailoring the PBG can improve the performance of PhC-based integrated optical devices, such as low-threshold lasers, Quantum cascade lasers, and high-Q photonic crystal nanocavities^13–18^. Recently, PBG has been looked at for improving solar cell efficiency and thermophotovoltaic applications^19,20^. Therefore, engineering the dispersion of PhCs and their gap map properties is essential and highly warranted.

Significant efforts have been applied over the past few decades to engineer the PBG in 2-D PhCs lattices^21–23^. For instance, one can explore bandgap properties by creating a degeneracy in the highest symmetric points of the Brillouin zone of PhCs. Some of the approaches to create degeneracy are identified as follows: (i) varying periodicity along one or two directions in the form of employing rectangular lattice^24^, (ii) usage of metallic/ dielectric rod in air medium and air rod in metallic/dielectric medium in hexagonal lattice^25,26^, (iii) varying the shape of PhC atom such as non-circular air holes in dielectric slab and rotation of constituent air holes^27^ and elliptical nanowires in air with different orientation of semi-major axis^28^, (iv) varying dielectric properties of PhC background and atom for change in pressure and temperature^29^, (v) reducing the symmetric properties of PhCs by combining two or more dielectric scattering objects in one-unit cell^30,31^, (vi) exploring complex unit cell structure such as vein networks and dielectric meshes^32^, (vii) by applying magnetic, mechanical, thermal, electrical and bio-chemical external stimulus to PhCs^33^, and (viii) novel 2-D tiles and tessellations such as Archimedean tilling, quasi crystal, hyper uniform disordered networks, continuous random networks and icosahedral quasicrystal^34–38^. All these methods are widely used to obtain large bandgaps in PhCs. There are also studies in self-assembled colloidal crystals stating that the size of PBG can be altered depending on the particle size and the internal structure^39^.

Recently, a new approach has been proposed to acquire a wide PBG by superimposing two different PhCs. In 2017^40^, Fei Meng et al. theoretically studied the evolution of PBG by superposing a transverse electric (TE) bandgap structure (vein structure) and a transverse magnetic (TM) bandgap structure (dielectric rods in an air medium). Further, topology optimization is applied to maximize the complete PBG. Similarly, Gómez-Urrea et al.^41^ considered two different PhCs of dielectric rod in an air medium, and by rotating PhC lattices one over the other, a new commensurable bilayer rotated square lattice PhCs with different permittivities are obtained. By changing the commensurable angle and the permittivity of the rods, the PBG of the resultant PhC can be tuned. This type of PhC lattice is known as the Bravais-Moiré photonic crystal lattice.

**Figure 1:**
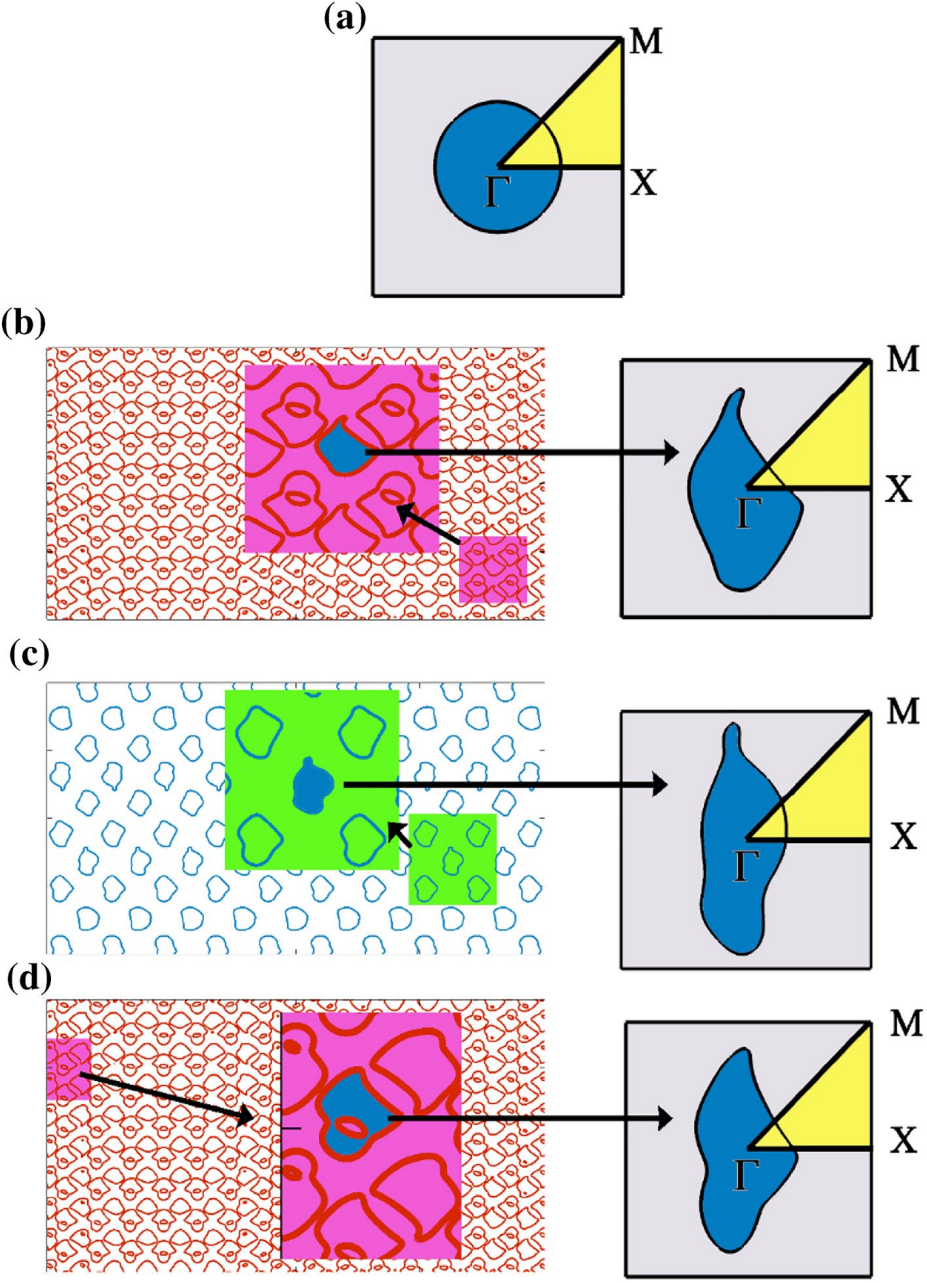
The square lattice PhC unit cell with the highest symmetry points is marked. (**a**) Circular rod (C), (**b**) L, (**c**) N, and (**d**) W obtained from the NM tiles for their corresponding coefficients (**b** and **d**) [1.25 2.52 2.27 1.96 2.54 2.83 44 2.74 74 2.76] and (**c**) [1 3 1 0.8 0.3 1.7 3 2.1 3 1.5].

In most of the above approaches, the realization of PhC is restricted to regular symmetric shapes arranged in square and triangular lattices. In this study, we have eased the restrictions on the shape/geometry of PhC’s atom with the help of non-Moiré (NM) tiles. For instance, Fig. 1 shows a few of the generated NM tiles. This pattern is referred to as an NM pattern because it is different from a Moiré pattern, which is created by superimposing two similar shapes at an angle to create a geometric pattern. Instead, in this work, we superimpose two different trigonometric functions to create ‘n’ different geometrical tiles. We derive and define unit cells from these NM tiles, arrange them in regular square and triangular lattices and solve their photonic bandstructures and gap map profiles. In the second case, we show how the NM tile acts as a kind of PhC. Through full-wave electromagnetic simulations, we also demonstrate the orientation-dependent frequency selective property of the proposed patterns. The proposed complex geometries can be fabricated efficiently using 3-D printing techniques. In this work, we also demonstrate the fabrication of one of the proposed geometries for microwave frequency regimes.

## Extraction of unit cell geometries from NM tiles

Figure 1 shows the unit cell of square lattice PhC consisting of proposed geometries obtained from the NM tile. The NM tiles are the contours of trigonometric functions represented by

$$z = e\sin \left( {x_{1} } \right) + f\cos \left( {y_{1} } \right)\left\{ \begin{gathered} x_{{\mathbf{1}}} \, = \, {\mathbf{asin}} \, \left( {{\mathbf{I}}x} \right) \, + \, {\mathbf{bcos}} \, \left( {{\mathbf{Jy}}} \right) \hfill \\ {\mathbf{y}}_{{\mathbf{1}}} \, = \, {\mathbf{ccos}} \, \left( {{\mathbf{K}}x} \right) \, + {\text{ d}}{\mathbf{sin}} \, \left( {{\mathbf{Ly}}} \right) \hfill \\ \end{gathered} \right.$$

where a, b, c, d, e, f, I, J, K, and L are coefficients. By varying these different coefficients, one can generate different tiles, as shown in Fig. 1. Two different periodic structures can be constructed from the proposed patterns. (i) One can employ the various shapes of the patches as PhC atoms in the regular square and triangular lattices. So that one can explore how the PhC bandstructure is modified concerning the conventional circular-rod PhCs. (ii) Since the obtained tiles are themselves periodic, they can act as a class of PhC. Therefore, we can use these obtained tiles as such to study for the PBG.

In the present study, we explore both tasks as follows: first, we select a specific pattern from the tile to construct a photonic atom as shown in Fig. 1b–d. The patterns shown in Fig. 1b and d correspond to the set of coefficients of [1.25 2.52 2.27 1.96 2.54 2.83 44 2.74 74 2.76] and [1 3 1 0.8 0.3 1.7 3 2.1 3 1.5], respectively. The choice of these coefficients is not specific, as one can generate an infinite number of tiles by varying ten different coefficients. However, we are interested in isolated patches with irregular geometries for achieving strong photonic bandgap. Hence, we varied the coefficients so that the periodicity and amplitude of the sinusoidal functions were modified to get the isolated contours. Hence, one can get ribbons, tiles, channels, and isolated patches by altering the coefficients. This work investigates how irregular patches open new avenues of photonic bandgaps. According to the contours of each asymmetric PhC atom shown in Fig. 1b–d, which resemble shapes like the liver (L), arbitrary (N), and wings (W), respectively, we designate them as L, N, and W patterns.

To compare the photonic bandstructure of the proposed patterns in square and triangular lattice arrangement, a circular rod of radius 0.252*a* (*a* is the lattice constant) arranged in square and triangular lattice is taken as a reference. In all the cases, the same filling fraction is ensured to reveal the role of structural anisotropy of the proposed geometries. The dielectric permittivity of the patterns is assigned as ε_r_ = 12.96 (equivalent to that of GaAs for optical frequencies), and the background is considered as air. In the unit cell diagram, Γ ($K_{x} = 0, \, K_{y} = 0$), X ($K_{x} = \frac{\pi }{a}, \, K_{y} = 0$), and M ($K_{x} = \frac{\pi }{a}, \, K_{y} = \frac{\pi }{a}$) are the highest symmetry points of the first irreducible Brillouin zone of the square lattice, where *K*_*x*_ and *K*_*y*_ are the components of the Bloch wavevector.

## Results and discussion: photonic bandstructures and gap maps of the proposed NM geometry-based 2-D PhCs

**Figure 2:**
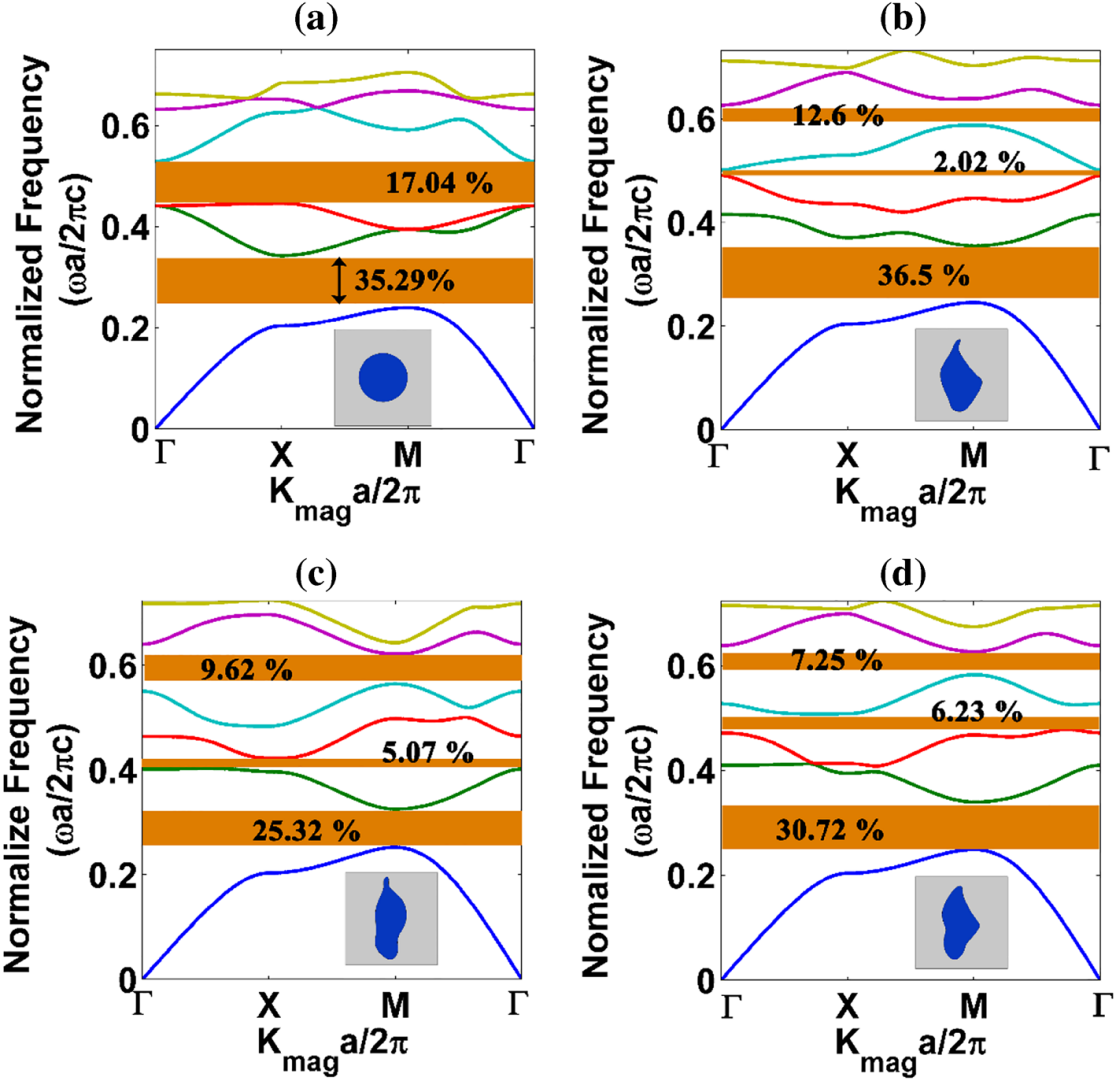
Bandstructure results of square lattice PhC Formed by anisotropic NM geometry patterns: (**a**) circle, (**b**) L, (**c**) N and (**d**) W structures.

For a square lattice PhC, bandstructure obtained along the highest symmetry points $(\Gamma ,\,X,\,M)$ of the irreducible Brillouin zone is shown in Fig. 2. The irreducible Brillouin zone (IRBZ) of a square lattice is shown in the upper right corner of Fig. 1a. It is reiterated that apart from circular rod PhC, bandstructure calculations for all other patterns must be calculated for the entire unit cell rather than the IRBZ due to the structural anisotropy of the patterns. For instance, we calculated the bandstructure of the proposed patterns for all the quadrants of square lattice PhCs, and they are given in the supplementary (Figs. S1 to S3). Here, for comparison purposes, only one quadrant of the first BZ is taken to highlight the difference between circular rod and proposed pattern-based PhCs.

Figure 2 shows the PBG results for the circular rod (Fig. 2a) and the other three proposed NM geometry-based rods [L geometry (Fig. 2b), N geometry (Fig. 2c) and W geometry (Fig. 2d) respectively] for TE polarization. A significant change in the PBG is observed for all the proposed NM patterns with respect to circular rod PhC. For instance, the second largest PBG is observed between III and IV bands in the case of a circular rod PhC, but for anisotropic NM structures (like Fig. 2b–d), the second largest PBG is observed between IV and V photonic bands. In Fig. 2d, the complete PBG is absent between II and III bands due to a Dirac-like point (a point at which two photonic bands merge) between Γ and X symmetric points in the band diagram. However, the W-like structure tries to open up the PBG in the X-M-Γ direction. It is also noted in Fig. 2d that a Dirac-like point is observed between Γ and X directions, whereas for a circular rod PhCs, a Dirac-like point is observed both at Γ and M points (Fig. 2a). Therefore, the PhC formed by structurally modified patterns allows one to alter the Dirac-like points.

Apart from Dirac-like points, one can also find a vast difference in the dispersion curve for the proposed patterns against the circular rod PhC. For example, if we consider the II band results obtained for NM PhCs, the flat band along the Γ-X direction is observed for all three anisotropic structures (Fig. 2b to d). The flat band nature represents the behaviour of EM waves to travel very slowly in the medium^42^ because the near-zero slope indicates near-zero group velocities and, hence, the exceptionally high value of group refractive index. Moreover, due to the flat band nature of PhCs formed by asymmetric patterns, many partial bandgaps are not observed as in the case of circular rod type PhC. Combining the above aspects, one can attain a unique anomalous dispersion characteristic using the proposed asymmetric patterns for different wave optics applications.

From the obtained bandstructure results, the PBG percentage is calculated. It is observed that primary PBG between I and II bands for conventional circular rod and L structure PhC is 35.29% and 36.5%, respectively. The second largest PBG for conventional circular rod PhC falls between the III and IV bands with a PBG percentage of 17.04%. However, the proposed asymmetric structures are advantageous to obtain bandgap at higher frequencies. It is true that by scaling circular rod PhCs, one can achieve PBG at any higher frequencies. However, the asymmetric patterns-based PhCs naturally open PBG at higher frequencies, so one can explore the structural anisotropy to get the PBG rather than scaling the structure. In addition to the primary bandgap, the highest bandgap percentage of 12.6% is observed for the L structure between IV and V bands.

**Figure 3:**
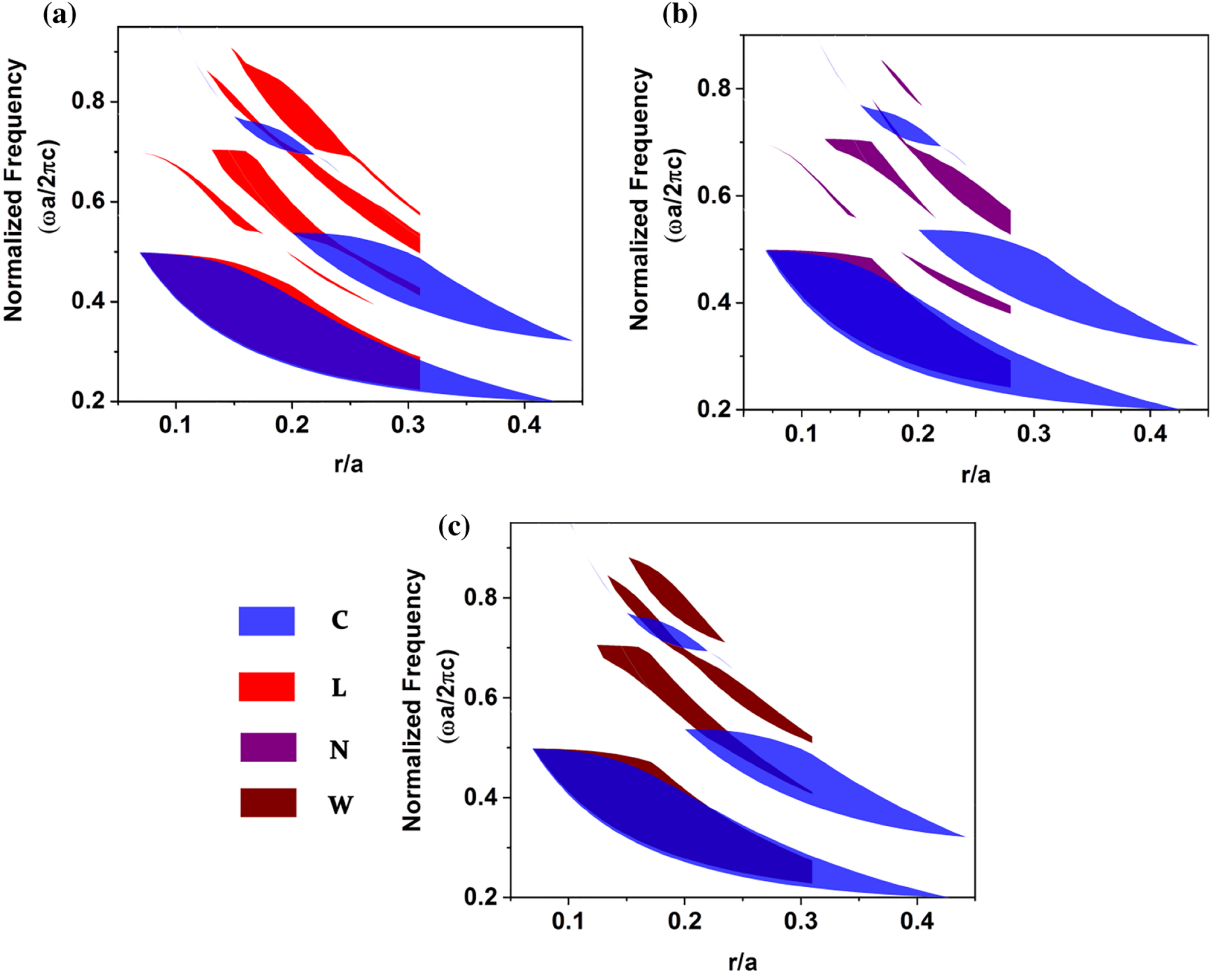
Gap map plot of a square lattice PhC formed by NM geometric patterns as a function of the normalized size of the photonic atom.

The gap map properties of the proposed square lattice PhC formed by NM geometric patterns are studied to understand the existence of large bandgap by plotting bandgap positions as a function of the rod’s radius for TE polarization. The gap map was studied through the dispersion diagram for a single unit cell of 2-D dielectric PBG for conventional and asymmetric structures. The dispersion diagram was obtained by varying the rod’s radius from 0.01*a* to 0.5*a* for conventional circular dielectric rods. For comparison, the area occupied by the unit cell of the proposed asymmetric rod was calculated and compared with the area occupied by the circular rod respective to its rod radius. For clarity, the gap map was plotted in the scale of the* r*/*a* ratio for normalized frequency. Figure 3 presents the gap map plot of square lattice PhCs formed by NM geometric patterns for TE polarization. It is significant to note from Fig. 3 that proposed PhCs exhibit new avenues of PBG at higher frequencies for lower filling fractions, and such gaps are absent in the case of circular rod PhC. Since the L pattern is highly anisotropic, some of the partial bandgaps observed for the L pattern are not presented in other structures. The bandgap size for proposed patterns is reduced when the filling fraction is increased. The reduced bandgap for increased filling fractions is attributed because the proposed patterns exceed the unit cell for higher filling fractions.

**Figure 4:**
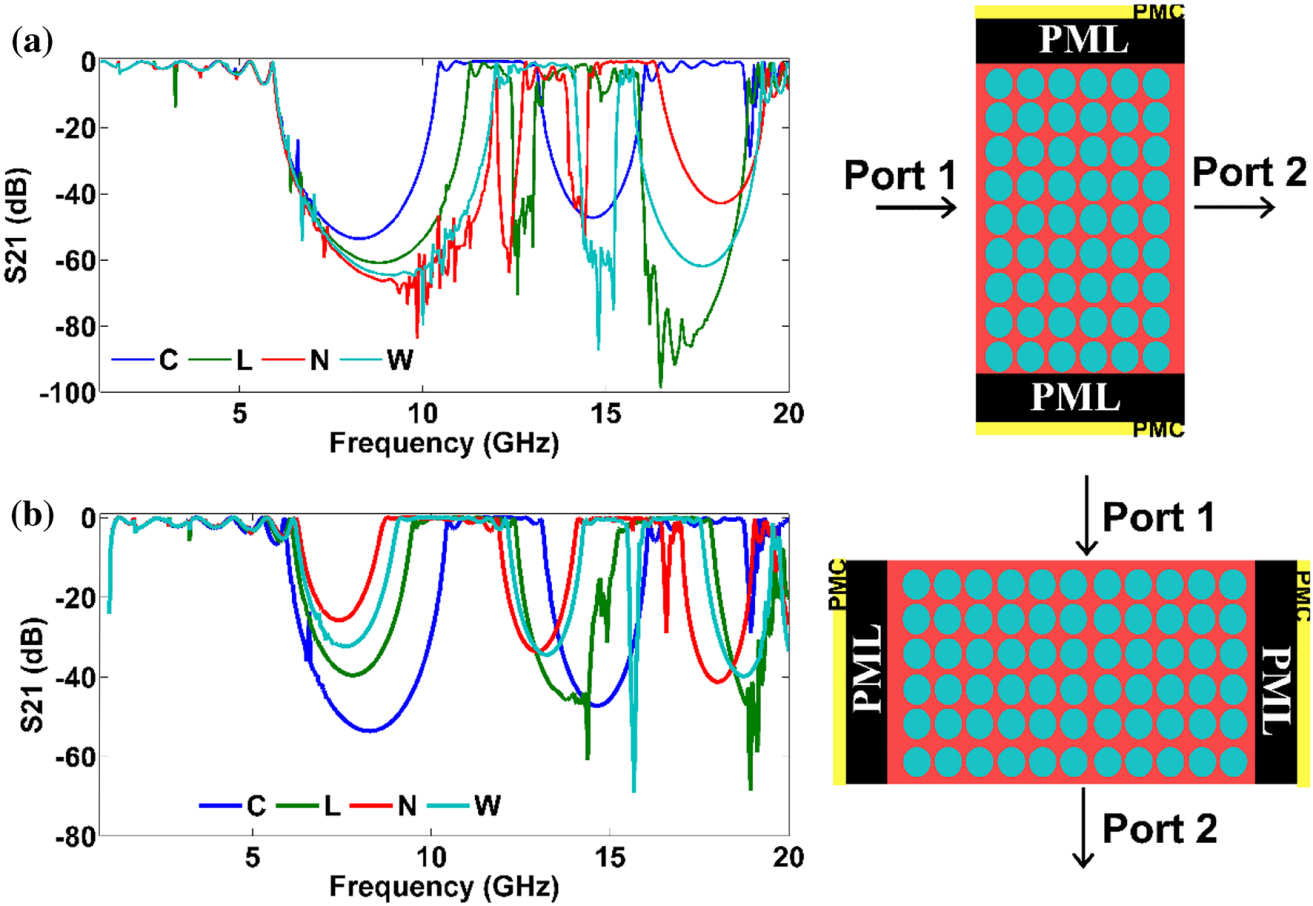
Transmission characteristics along normal incidence and $\Gamma - X^{\prime}$ direction is shown in (**a**) and (**b**). The schematics explain the direction of incidence along the port for TE polarization with boundary conditions used to calculate the transmission spectrum.

The transmission characteristics of the PhCs formed by the proposed NM patterns were studied for TE polarization and are given in Fig. 4. The transmission characteristics of the system were analyzed by choosing the same filling fraction (area occupied by unit cell ~ 1.99 cm^2^) for all NM patterns. Since the proposed pattern's symmetry is different for $X{\text{ and }}X^{\prime}$, the transmission parameter of NM pattern PhC is computed for both symmetric directions (i.e.$\Gamma - X$, and $\Gamma - X^{\prime}$). It is found that the bandgap of the proposed PhCs is wider for normal incidence along $\Gamma - X$ the direction. However, $\Gamma - X^{\prime}$ the bandgap decreases for anisotropic structure along the direction, as shown in Fig. 4b. In this case, the bandgap decreases due to the filling fraction ratio, i.e., along $\Gamma - X^{\prime}$ the direction, the filling fraction is higher for the proposed structure than the conventional system.

The bandstructure result (Fig. 2) shows that the L structure possesses a broader bandgap than conventional geometry, whereas other proposed geometry yields a lower bandgap width. However, if we observe the transmission plot (Fig. 4a), the width of the stop band is more comprehensive for the asymmetric structure. Therefore, the proposed asymmetric structure is more beneficial to achieve a wider bandgap, at least for normal incidence. Another exciting factor inspired by Fig. 4b is that the PBG can be tuned by changing the orientation of the NM pattern.

**Figure 5:**
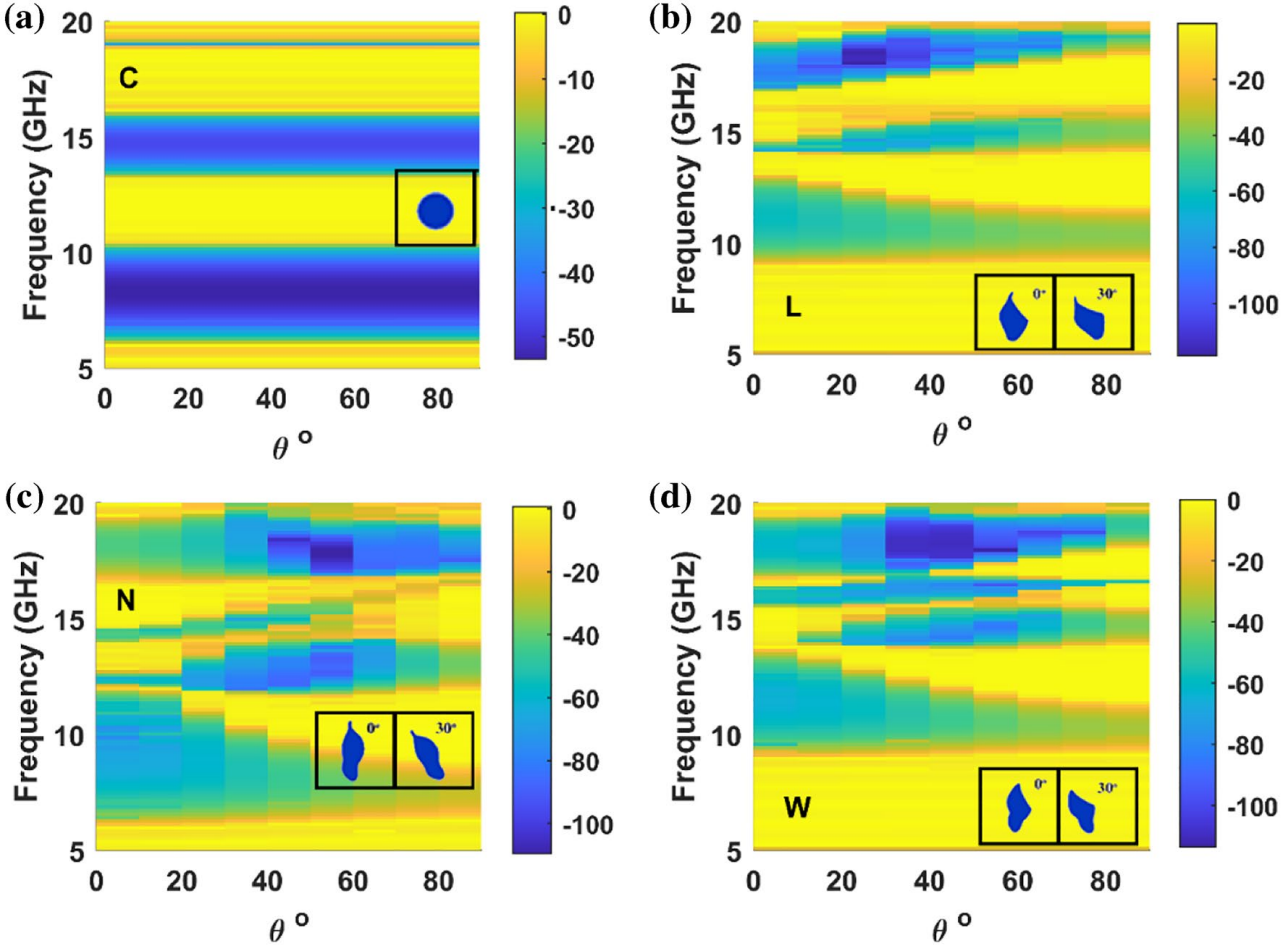
Transmission plot along different orientations of the unit cell along normal incidence for TE polarization. (**a**) circular rod, (**b**) L rod, (**c**) N rod and (**d**) W rod respectively.

To study the orientation of NM pattern in the PBG’s strength, the unit cell of the proposed geometry was rotated as shown in the inset of Fig. 5. As in the previous case, each PhC with 7 × 15 array of atoms is taken and perfectly matched boundary layers are employed along the top/bottom layers as shown in Fig. 4. Since the circular rod is highly symmetric along all the directions, the transmission is independent of orientation (Fig. 5a). However, for the proposed NM pattern-based PhCs, the orientation of patterns significantly alters the transmission characteristics. For all asymmetric structures, an increase in the orientation angle of the unit cell decreases the primary PBG width, as shown in Fig. 5b–d. Similarly, the bandgap's depth (rejection level) alters with respect to the orientation of the NM patterns. When we compare the rejection ratio of symmetric structure (Fig. 5a) and asymmetric structure (Fig. 5b–d), the asymmetric structure possesses a higher rejection ratio at a higher frequency range where PBG is absent in those ranges for symmetric structure. For instance, symmetric geometry does not possess a PBG region when we look at frequencies between 17 and 19 GHz. In contrast, asymmetric structures open the PBG region with high rejection ratios, and it decreases with the increase in the orientation of the unit cell for L and W structures. Also, one can notice many higher order bandgaps (distinct from primary and secondary bandgap as of C) and higher frequency bandgaps (new avenue of PBG when compared to C structure) from Fig. 5.

### Bandstructure results for triangular lattice PHCs formed by NM geometries

**Figure 6:**
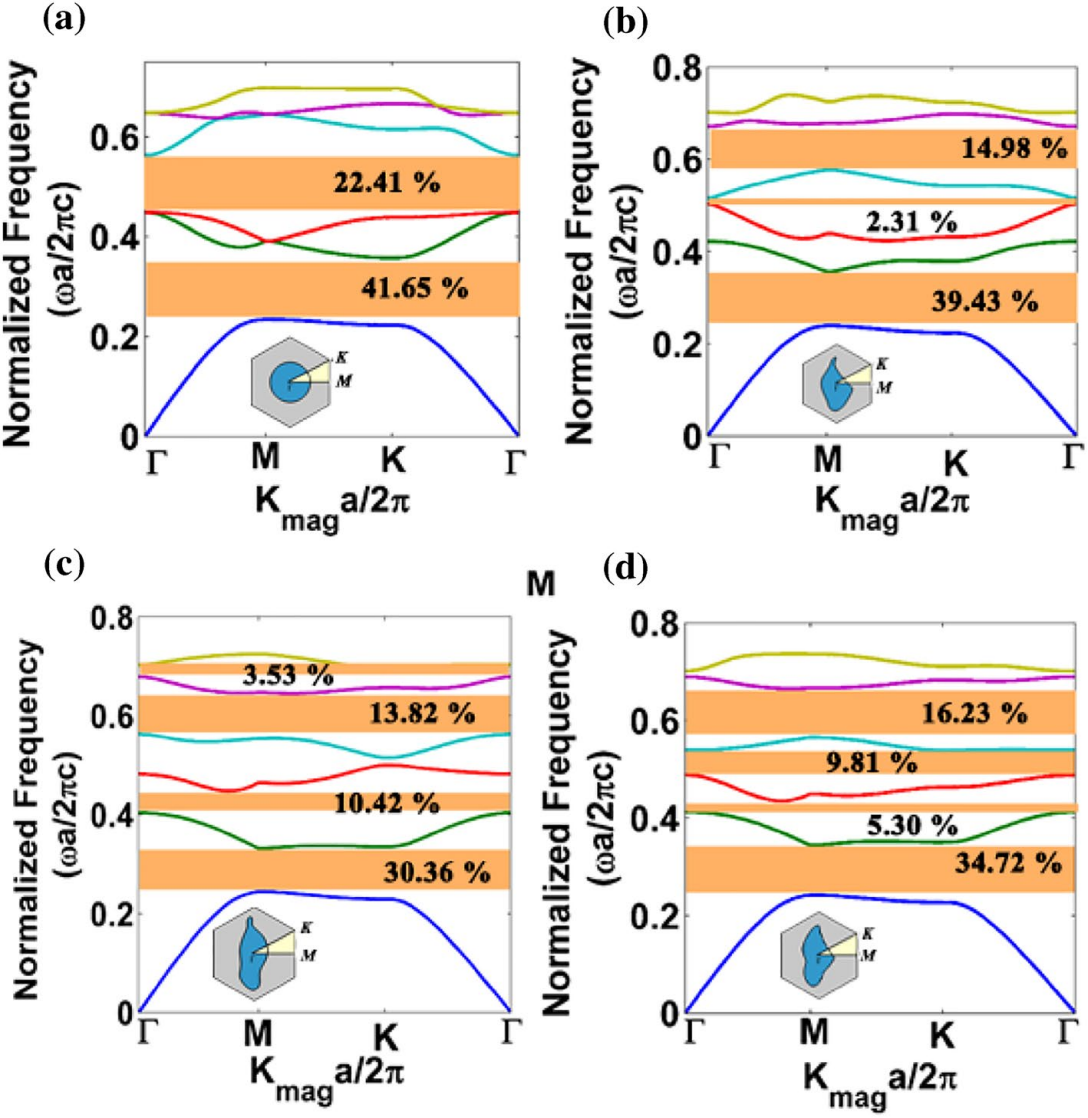
Bandstructure results of dielectric rod arranged in a triangular lattice with air background (**a**) Circular rod and (**b**–**d**) asymmetric rods corresponding to L, N and W, respectively.

Since the triangular lattice arrangement provides higher connectedness among the photonic atoms than the square lattice, the photonic bandstructure for the circular rod and the proposed NM pattern-based triangular PhCs are plotted in Fig. 6 for TE polarization. Two complete PBG regimes are observed for a circular rod triangular PhC (Fig. 6a). In the case of the proposed asymmetric patterns-based triangular lattice PhCs, new complete PBG regimes are witnessed at higher band frequencies. Though the width of complete PBG is thin due to structural anisotropy, one can achieve complete PBG between any photonic bands using the proposed asymmetric patterns. The PBG percentage obtained for each proposed NM pattern-based triangular PhC is shown in Fig. 6.

The primary PBG percentage of circular rod triangular PhC is found to be 41.65% for TE polarization, whereas, in the case of asymmetric structure, the primary PBG was calculated to be 39.43%, 30.36%, and 34.72% for L, N, and W structure respectively. Also, it is notable that PBG is present between each band for proposed structures, which is absent for the conventional structure. Henceforth, the proposed asymmetric unit cell in a triangular lattice will be an excellent candidate for attaining PBG between each photonic band. The lowest PBG was calculated to be 0.25% between the V and VI bands of the L structure. In the case of PBG at higher frequencies, the W pattern shows 16.23% of PBG between the IV and V bands. It suggests the suitability of extending the same PhC for high-frequency applications. Since the triangular lattice PhCs exhibit bandgap for both the polarizations, bandstructure calculations for the asymmetric pattern-based PhCs are also extended for TM polarizations, and gap-to-midgap ratio percentage details are presented in Supplementary. In the case of TM polarization, N structure shows 7.74% (II-III bands) and 16.65% (III-IV bands) of PBG. Other asymmetric patterns show thin PBG for TM polarization.

**Figure 7:**
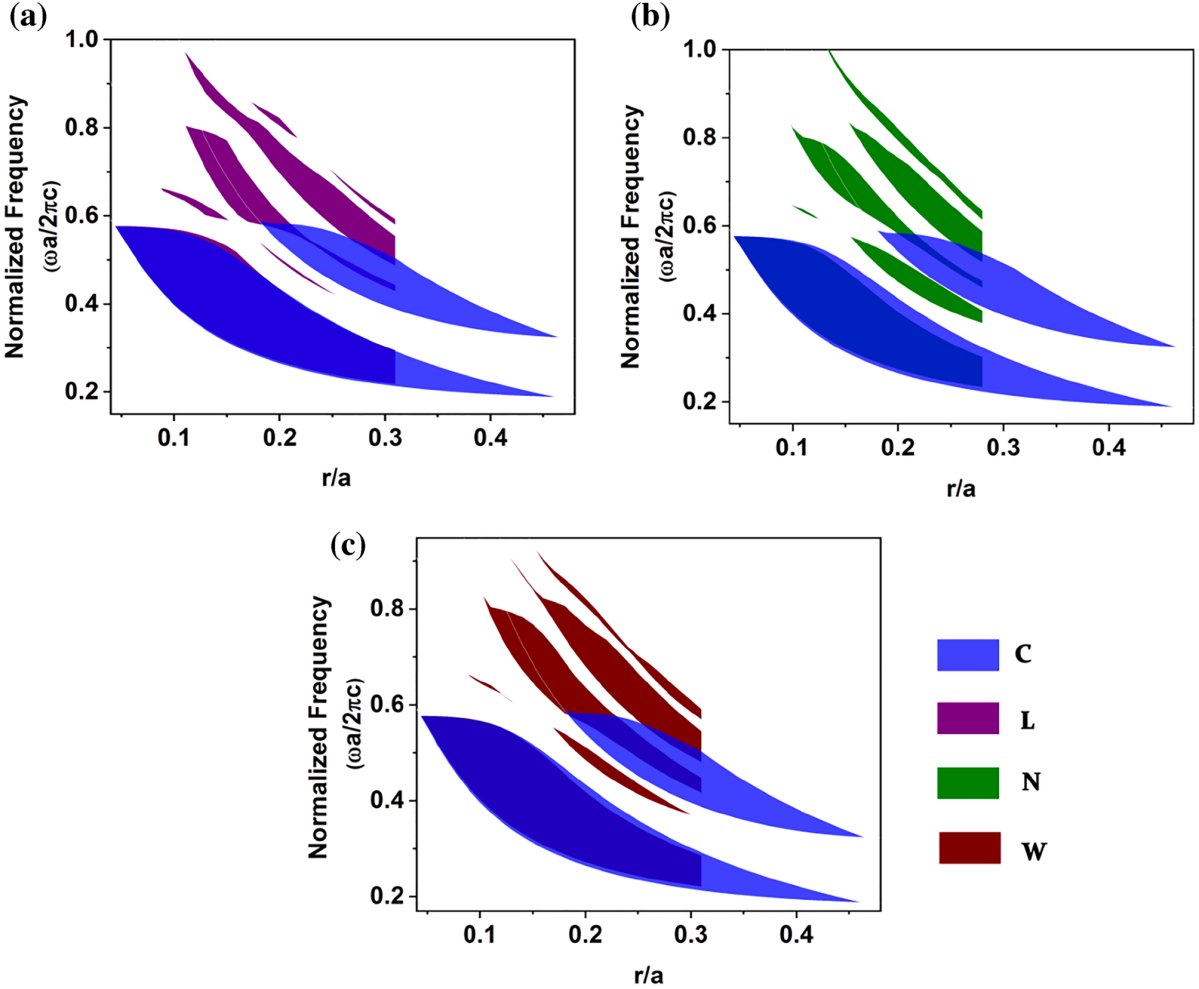
TE gap map profile of a 2-D triangular lattice PhCs for (**a**) circular and L, (**b**) circular and N and (**c**) circular and W patterns. Here, circular PhC is taken as a reference and compared with the gap map of every asymmetric pattern PhC.

The gap map profile of triangular lattice for TE polarization shows that most of the higher band PBG at a lower *r*/*a* ratio are witnessed only for the proposed asymmetric patterns. Thus, the proposed asymmetric pattern-based PhCs are identified as potential candidates for exhibiting PBG at higher frequencies for low-filling fractions, as shown in Fig. 7.

**Figure 8:**
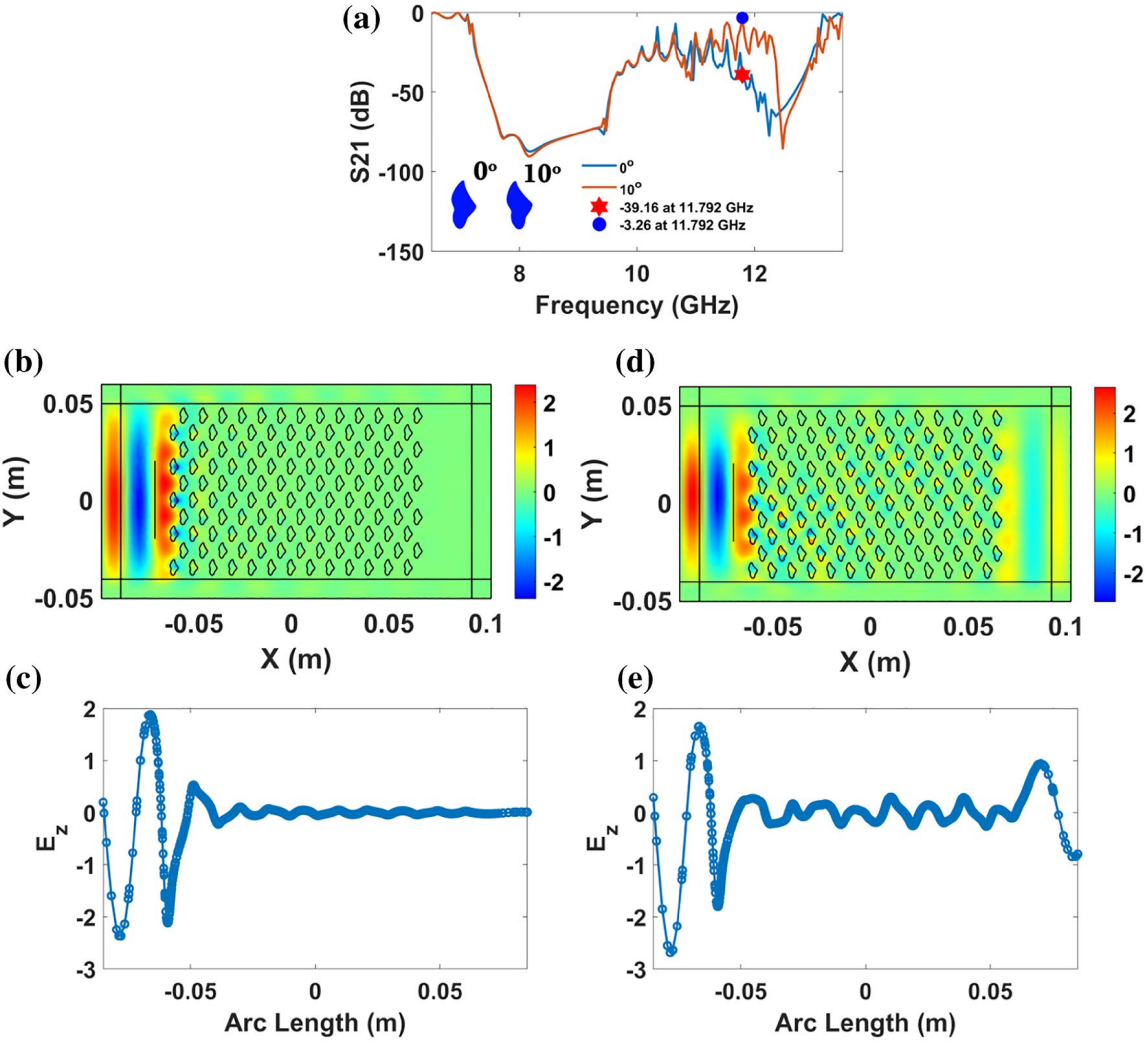
(**a**) Transmission (S21 (dB)) spectra of W pattern triangular PhC along ΓM direction for orientation angles 0° and 10° of W-pattern (atom orientation is shown in the inset) for normal incident TE polarization. (**b**) and (**c**) E_z_ field map and electric field scanning profile of W-pattern’s orientation angle 0° for normal incident TE wave at 11.792 GHz, respectively. (**d**) and (**e**) E_z_ field map and electric field scanning profile of W-pattern’s orientation angle 10° for normal incident TE wave at 11.792 GHz, respectively.

Since the proposed patterns are asymmetric, the orientation of the PhC atom will strongly influence the transmission characteristics of the proposed PhCs. In the previous section of the manuscript, the effect of orientation of asymmetric PhC atom for square lattice is carried out. Similar studies are also extended to triangular lattice-based asymmetric patterns. Particularly in Fig. 8a, transmission loss is recorded for W-pattern-based triangular PhC for two different orientations, 0° and 10°, by rotating the orientation of the unit cell. For instance, when the orientation angle of the W-pattern is 0°, the transmission loss observed at 11.792 GHz is 39.16 dB (i.e. the PhC strongly prohibits the EM wave from passing through it). This is evident from the E_z_ field map and electric field scanning profile in Fig. 8b and c for normal incident TE wave at 11.792 GHz, respectively.

On the other hand, when the orientation angle of the W-pattern is 10°, the transmission loss observed at 11.792 GHz is 3.26 dB (i.e. this time, the PhC allows light with transmission efficiency of 68.71%). The low transmission loss is verified from the E_z_ field map and electric field scanning profile in Fig. 8d and e for normal incident TE wave at 11.792 GHz, respectively. Though the transmission loss is considerable, the observed feature indicates the filtering functionality of the proposed asymmetric pattern-based PhC for two different orientations. Thus, the orientation of the PhC atom provides dynamical control and reconfigurability of filtering response in photonic circuits.

### NM tile itself as a PhC

**Figure 9:**
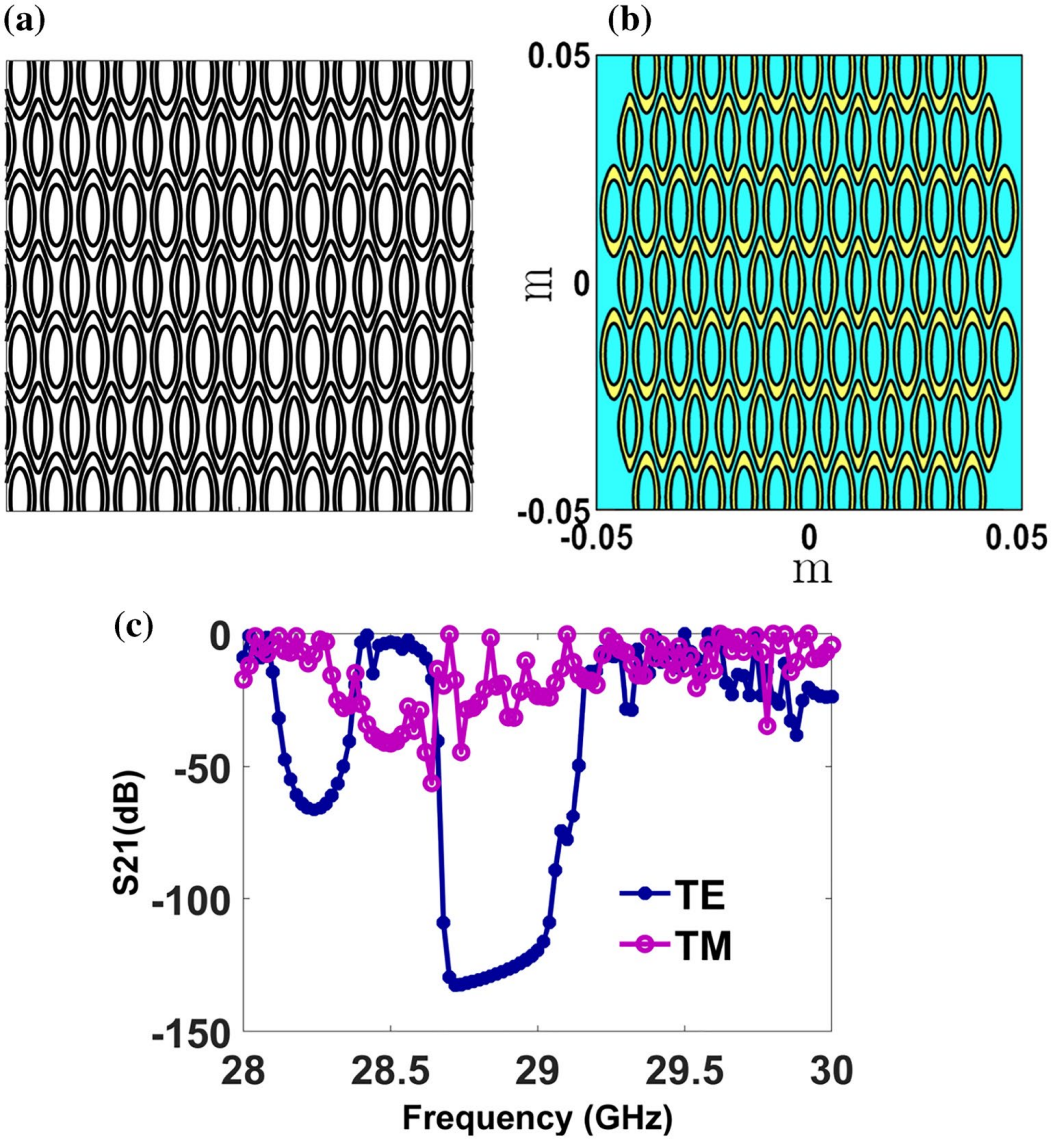
(**a**) NM tiles obtained for coefficients [0 1 2 0 1 1 1 1 2 1] (**b**) The PhC design drawn out of the NM tile shown in (**a**) with microwave length scales and (**c**) transmission loss spectra of NM tile PhC for normal incident TE and TM polarized waves.

Until this section, we have explored 2-D square and triangular PhCs formed by asymmetric patterns derived from NM tile (i.e. a specific contour of a trigonometric function is selected from the contour plot of Eq. (1) and PhC is built out of it). However, one can directly employ the NM tile pattern for controlling the EM radiation at microwave, THz, and visible frequencies. To demonstrate the concept of using the NM tile itself as a PhC, we consider 2-D contours obtained from the NM equation for the coefficients [0 1 2 0 1 1 1 1 2 1] as shown in Fig. 9a. The obtained NM patterns represent hollow elliptical structures in a periodic manner with two different filling fractions occupied by each elliptical structure. In this contour plot, a PhC is created by a dielectric (ε_r_ = 12) with the scheme shown in Fig. 9b. The PBG studies for the proposed tile are studied in the microwave length scale for a normal incident TE and TM polarized wave and their transmission loss spectra are plotted in Fig. 9c. From the Fig. 9c, the PBG was witnessed for both TE and TM polarizations. In the case of TE polarization, strong PBG bands are in the range of 28.08 GHz to 28.4 GHz (with a zero-transmission level of around 65 dB) and 28.6 GHz to 29.2 GHz (with a zero-transmission level of around 120 dB). In the case of TM polarization, a PBG in the range of 28.28 GHz to 28.7 GHz is observed with a zero-transmission level of around 40 dB. These aspects indicate that the NM tile itself acts as a PhC. Therefore, the realized NM tile is anticipated to help control EM signals in textile and wearable photonic circuits.

The photonic bandgap properties investigated in this work is compared with other reported 2-D PhCs, as in Table 1. Comparing previous works, the proposed NM tile-based PhCs provide additional freedoms (shape, different orientations, varying filling fraction) in controlling the PBG and dispersion properties. For instance, PBG at higher frequencies, tuneable bandgap depending upon the orientation of the unit cell and using the entire tile as a PhC can be employed for the emerging textile and reconfigurable photonic applications across the EM spectra.

**Table 1:** Main functionalities of the 2-D periodic structures reported.

Dimension	Geometries	Lattice	Year of publication [ref. no]	Achieved functionalities
2-D	Gyroid structures		2015^21^	Optical activity Dichroism
2-D	New geometries	Square	2015^22^	Self-collimation
2-D	Circle/square/hexagonal	Square/hexagonal	2010^23^	Large area gap map
2-D	Circle	Rectangular	2015^24^ 2017^25^	New bandgap regions Flat band
2-D	Circle	Hexagonal/honeycomb	2019^26^	Large bandgap Dirac-like points Beam steering
2-D	Reducing symmetry of the geometry	Honeycomb/triangular	2008^29^	Large complete PBG
2-D	Supercell	Honeycomb	2017^31^	Complete PBG
2-D	Complex unit cell	Square/honeycomb	2012^32^	Large complete PBG
2-D	Circle	Square/hexagonal	2014^33^	Tuneable PBG
2-D	Archimedean tiles		2017^34^	Self-collimation Negative refraction
2-D	Tessellation		2009^36^	Large complete PBG
2-D	Quasi crystals		2014^37^	Deep photonic bandgap
2-D	Self-assembled colloidal crystal		2021^39^	Tuneable PBG
2-D	Superposition of TE and TM structures		2017^40^	Complete PBG
2-D	Bravais–Moiré		2020^41^	Wider bandgap
2-D	Non-Moiré tiles (Contours of trigonometric functions)		In this work	(i) New bandgap regimes, including partial bandgaps with respect to circular-rod PhCs (ii) Direction-dependent PBG (iii) Tile itself a PhC (iv) 3-D printing fabrication

## Fabrication of proposed NM patterns using 3-D printing

The asymmetric PhC rod was printed using a 3-D printer based on the principle of fused deposition modelling, which works based on additive manufacturing techniques. For this work, a 3-D PhC model is constructed using COMSOL Multiphysics software where 3-D modelling space is considered and the geometry is built within a planar domain and then extruded along the z-axis to form a 3-D object. 3-D modelled object information is fed to the 3-D printer in the form of g-code with other printing parameters, as mentioned in Table 2.

**Table 2:** 3-D printing Fabrication parameters of CPLA-based L-shaped rod.

3-D printing parameters	Values
Printing temperature	210 °C
Bed temperature	60 °C
Print speed	50 mm/s
Infill percentage and pattern	100 and line
Layer height	0.2 mm

**Figure 10:**
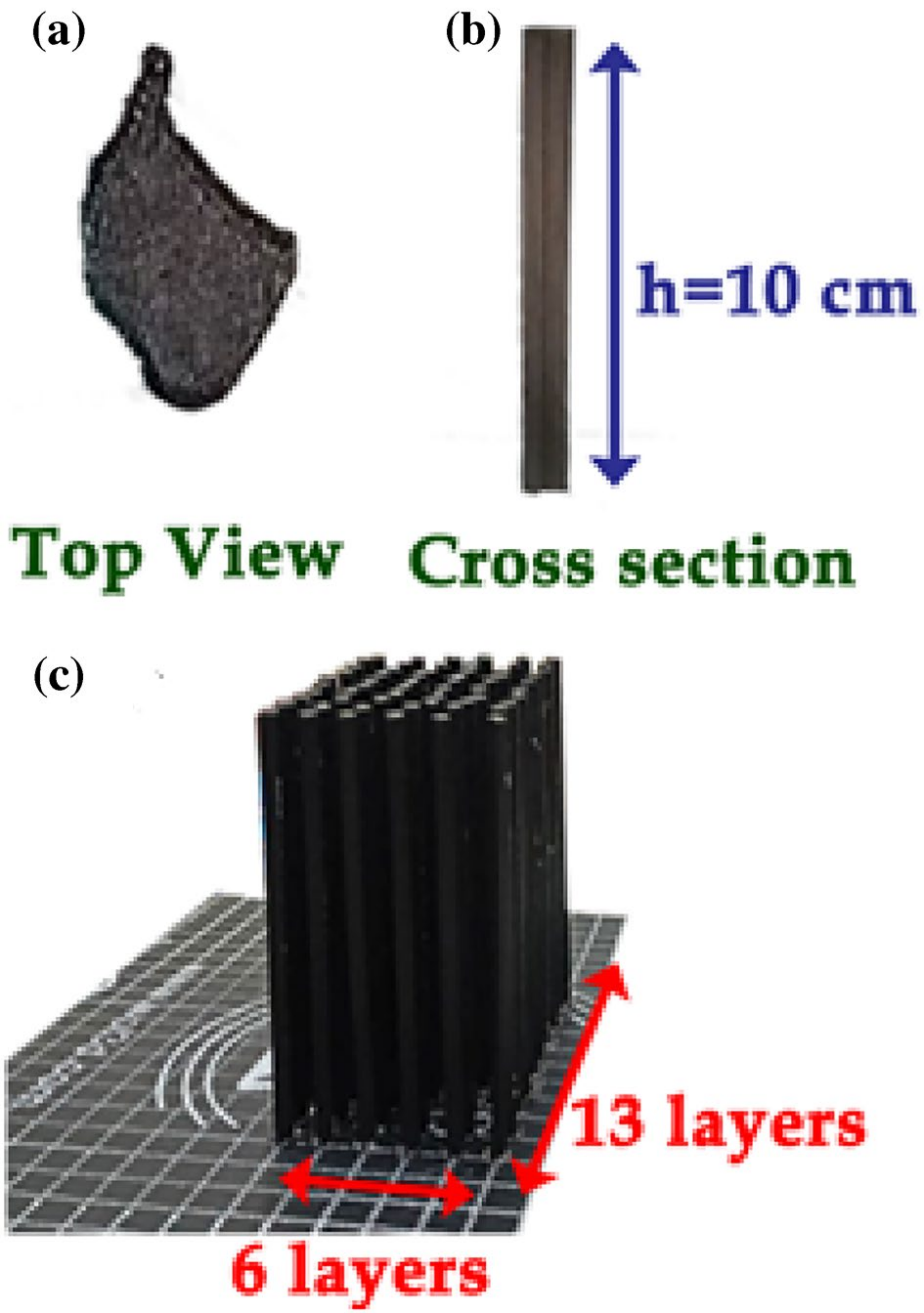
(**a**) top view and (**b**) cross-section of single PhC fabricated rod with height h = 10 cm and (**c**) Fabricated PhCs rods with 6 × 13 layers.

During this fabrication, conductive polylactic acid (CPLA) was chosen to build the PhC atom, depending on the dielectric strength of different types of PLA, as reported by Huber et al*.*^43^. From^40^, the experimental dielectric constant value of CPLA is found to be an average of 6.84 with a high loss tangent ($\tan \delta$) of 0.493 with a volume resistivity of 115 Ohm/cm at 9–10 GHz. In this work, asymmetric PhC made of an L-shaped rod is printed with the same parameters used to calculate bandstructure. The fabricated single PhC rod is shown in Fig. 10a and b with top and cross-sectional views, respectively. The periodic arrangement of PhCs is glued on a 3-D printing bed using a hot glue stick, and the fabricated CPLA is given in Fig. 10c. Thus, we successfully fabricated the proposed NM tiles pattern-based PhC for microwave regimes.

**Figure 11:**
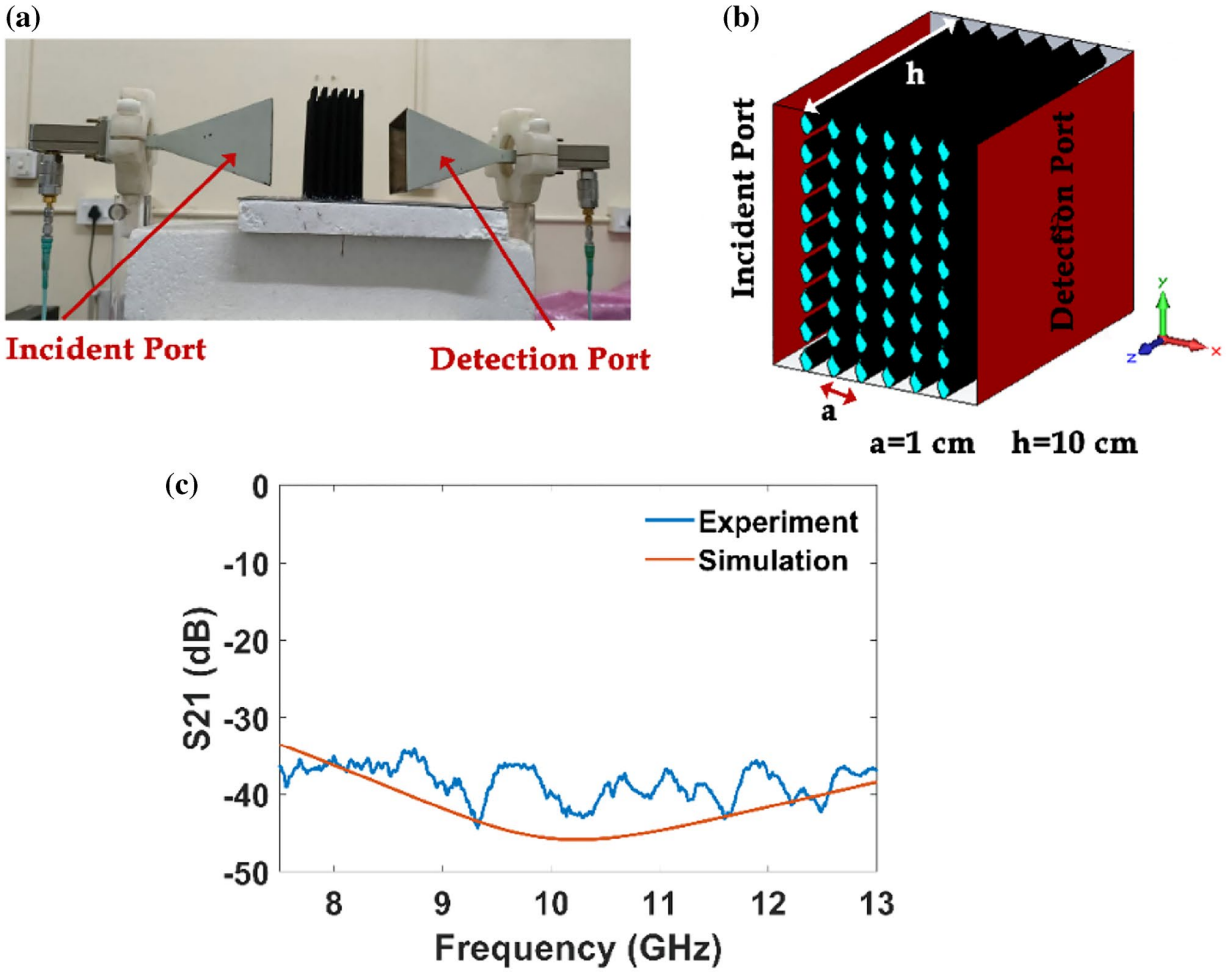
(**a**) Experimental setup with two horn antennas as transmitter and receiver connected to the vector network analyzer. (**b**) the geometrical setup used for numerical calculation, and (**c**) transmission spectra of CPLA PhC obtained from both simulation and experiment.

The transmission characteristics of the fabricated asymmetric L-shaped rod square lattice PhC are studied at microwave length scale. For the experimental work, we chose the periodicity of the PhC to be 1 cm. The transmission characteristics of the CPLA-based L-shaped rod PhC are measured using a Vector Network analyzer (N5230A) using standard gain X-band horn antennas (7 GHz to 13 GHz) with the setup shown in Fig. 11a. Similarly, numerical calculation of an L-shaped rod PhC array of 6X13 is taken where each rod has a finite height of 10 cm, as shown in Fig. 11b. The top/bottom and left/right layers of the PhC geometry are specified with perfect electric conducting and perfect magnetic conducting boundary conditions, respectively. Waveguide ports are set for transmission and reflection characteristics. Figure 11c shows the experimental and numerical transmission loss spectra of the CPLA-based L-shaped rod PhC. It is found that the transmission loss of CPLA PhC is over 30 dB, and it indicates the existence of strong attenuation for normal incident TE wave. High transmission loss is mainly due to the material property, i.e. CPLA material has very high dielectric loss. In the supplementary figure, the transmission loss for various dielectric losses is computed numerically. Since we aimed to show how to fabricate a proposed asymmetric NM tile pattern, we relied on low-cost CPLA material. However, currently, we fabricate other interesting NM tile patterns using low-dielectric loss 3-D printing materials.

## Conclusion

To conclude, we have explored the photonic bandstructure calculations for 2-D PhCs formed by asymmetric patterns derived from NM tiles. Using FEM methodology, PBG calculations are successfully calculated for 2-D PhCs with square and triangular lattices, and results are validated for newly realized asymmetric pattern-based PhCs. As a result, it was found that asymmetric pattern-based PhCs open new PBG for both square and triangular lattices with respect to circular rod-based conventional PhCs with strong zero-transmission levels when compared with the conventional circular rod PHCs. The bandstructure of asymmetric pattern-based PhCs consists of flat bands, which are helpful for slow light applications. Also, it is essential to note that the orientation of asymmetric patterns strongly influences the PBG characteristics. Based on this feature, a reconfigurable filtering functionality is demonstrated for W-pattern PhC. We have also demonstrated numerically how the NM tile itself acts as a kind of PhC. We also successfully fabricated a few of the proposed NM patterns using the 3-D printing technique using conductive PLA material. We are extending the 3-D printing technique to fabricate other patterns with low dielectric loss materials. Our result opens further avenues in molding the light flow in the domains of reconfigurable optics, light confinement and wave optics applications.

## Methodology: photonic bandstructure calculations and benchmarking

The photonic bandstructure (PBS) calculations for the proposed geometries in square and triangular lattices are computed by solving Maxwell’s wave equation as an eigenvalue problem^44^. Usually, PBS are performed through planewave expansion (PWM) method using open-source solvers MPB (MIT Photonic Bands)^44^ or MATLAB codes. In the PWM method, the eigenvalue problem is constructed in terms of magnetic field strength vector subjecting to the constraints (i) $\nabla \cdot \vec{H}\left( {\vec{r}} \right) = 0$, (ii) $\nabla \cdot \varepsilon_{r} \vec{E}\left( {\vec{r}} \right) = 0$(source-free regime) and (iii), $\left\langle {\vec{H}_{n} \left( {\vec{r}} \right)\left| {\vec{H}_{m} \left( {\vec{r}} \right)} \right.} \right\rangle = \delta_{nm}$ (orthogonality of modes). All the physical parameters are expanded in terms of Bloch-wave functions (Planewave vectors spanning over integral multiples of reciprocal lattice vectors). The accuracy of the solutions of eigenfunctions (modes) and eigenvalues (mode frequencies) depends on the number of planewaves. Even though the PWM method using MPB or MATLAB is robust and accurate, implementing complex geometries in these solvers is difficult. Instead, we solve PBS calculations of the proposed geometries using a commercial EM solver COMSOL RF Module through an eigenvalue problem.

In COMSOL, any 2-D patterns can be drawn or imported from MATLAB. In our work, we imported the patterns shown in Fig. 1 from MATLAB into the COMSOL RF module and performed the eigenvalue problem. The constraints and approximations used in the PWM method also apply to the COMSOL solver. However, COMSOL's eigenvalue problem is based on the finite-element method (FEM), in which the unit cell geometry is discretized into extra fine meshes. Hence, the number of mesh elements influences the mode patterns accuracy and frequencies. In COMSOL, both TE and TM bandstructure can be separately solved for two different calculations: (i) one for out-of-plane vector (E_z_ mode, TE Polarization (in MPB, this definition is designated as TM polarization) and another one for in-plane vector (E_y_ mode, TM polarization).

The bandstructure is solved by searching the eigenvalues for the range of Bloch wavevectors around ΓX, XM and ΓM directions (i.e. around the edges of irreducible BZ boundaries). Similarly, for a hexagonal lattice, eigenvalues are solved for the range of Bloch wavevectors around ΓM, MK, and ΓK directions, where Γ ($K_{x} = 0, \, K_{y} = 0$), M ($K_{x} = 0, \, K_{y} = \frac{2\pi }{{a\sqrt 3 }}$), and K ($K_{x} = \frac{2\pi }{{3a}}, \, K_{y} = \frac{2\pi }{{a\sqrt 3 }}$) are the highest symmetry points of the irreducible BZ of the hexagonal lattice.

For benchmarking the results, we first solve the known PhC case (square and triangular lattice PhC formed by circular rods arranged in air background) using both PWM (using MPB solver) and FWM (using COMSOL solver). The photonic bandstructure solved by both methods is given in supplementary, and they are in excellent agreement. To compare the accuracy and reliability of COMSOL calculations, we tabulated the PBG percentage value in supplementary, which is computed as the percentage of gap to mid-gap ratio $${\text{PBG}}\left( \% \right) = \frac{{\omega_{2} - \omega_{1} }}{\Delta \omega }, \, \Delta \omega = \frac{{\omega_{1} + \omega_{2} }}{2}$$$$\omega_{1} {\text{ and }}\omega_{2}$$, corresponding to lower/upper photonic band edge frequencies of the PBG, respectively. It is found that PBG values from two different methods (FEM and PWE) are in excellent agreement with each other within the error limit of 0.5%. Thus, the benchmark studies prove the reliability of FEM-based bandstructure calculations using the COMSOL RF Module. Therefore, one can rely on COMSOL to obtain the bandstructure for complex photonic geometries.

### Supplementary Information

Supplementary Information.

## Data Availability

Data underlying the results presented in this paper are not publicly available at this time but may be obtained from the corresponding author (Natesan Yogesh; yogesh@nitc.ac.in) upon reasonable request.
