# Supplementary material for: Accessing new avenues of photonic bandgaps using two-dimensional non-Moiré geometries
Source: Sci Rep. 2023 Oct 10;13:17077. doi: 10.1038/s41598-023-44385-z (PMC10564743; doi:10.1038/s41598-023-44385-z)
Supplement: Supplementary file 1 — Supplementary Information. [file 41598_2023_44385_MOESM1_ESM.docx]

Supplementary Information: Accessing New Avenues of Photonic Bandgaps Using Two-Dimensional Non-Moiré Geometries

R. Rachel Darthy^1^, C. Venkateswaran^1^, V. Subramanian^2^, Zhengbiao Ouyang^3^ and N. Yogesh.^4,a)^

^1^Department of Nuclear Physics, School of Physical Sciences, University of Madras, Chennai-600025, India

^2^Microwave Laboratory, Department of Physics, Indian Institute of Technology Madras, Chennai-600036, India

^3^Terahertz Technical Research Center, College of Physics and Optoelectronic Engineering, Shenzhen University, Shenzhen-518060, China

^4^Department of Physics, National Institute of Technology Calicut, Kozhikode-673601, Kerala, India

^a)^Corresponding author: E-mail address: [yogesh@nitc.ac.in](mailto:yogesh@nitc.ac.in)

**I. Bandstructure Calculations Over All Quadrants of First Brillouin Zone (BZ)**

The proposed patterns are asymmetric and irregular and therefore, it is essential to compute the bandstructure not only for the irreducible Brillouin zone (IBZ) but also for the overall quadrants of the first BZ. Figure. S1 to S3 show the bandstructure calculations in all eight quadrants of L, N, and W PhC structures, respectively.


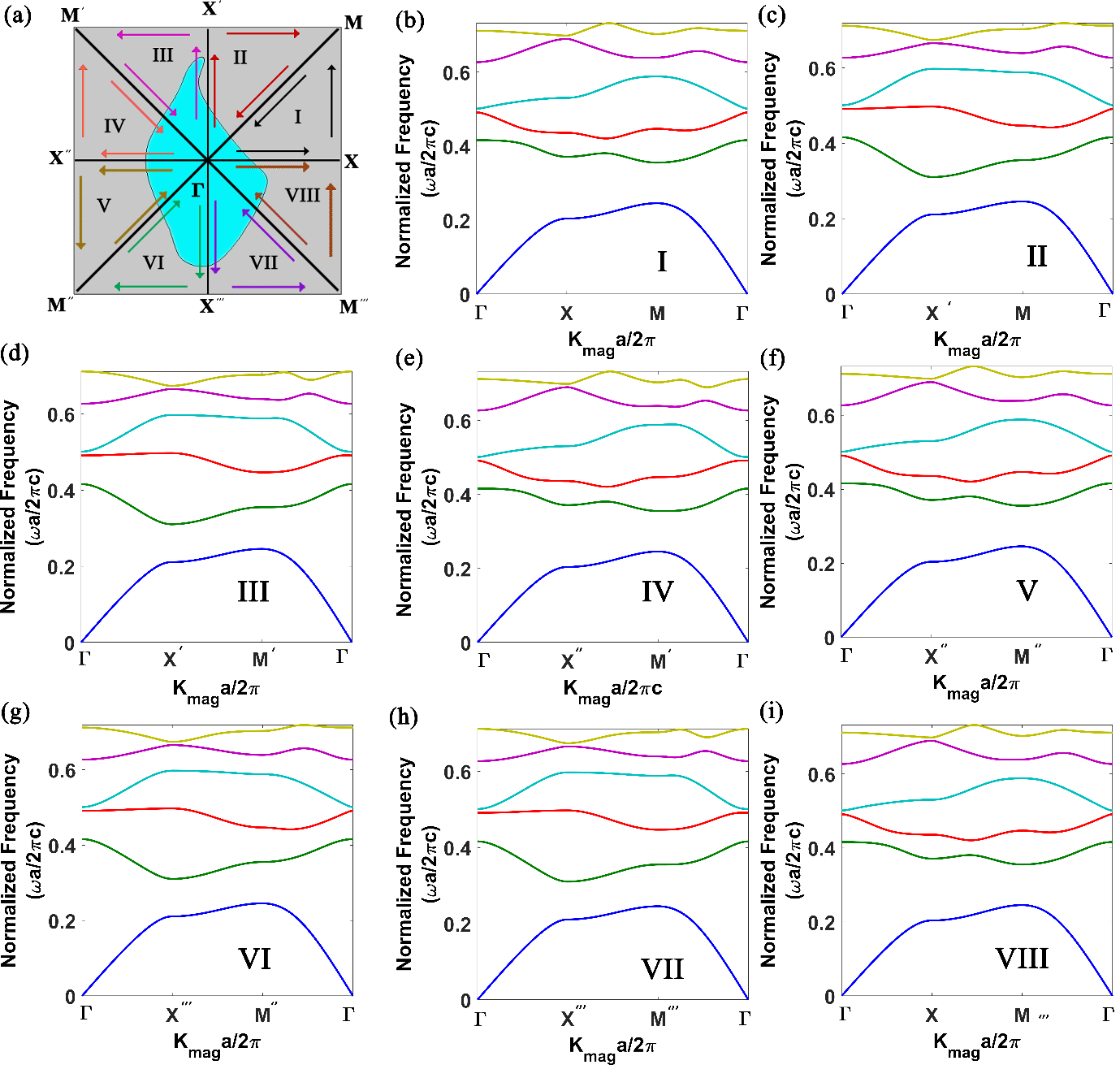


Fig. S1. Bandstructure obtained for the entire zone by considering eight different quadrants of the unit cell shown in (a). (a) L-structure square lattice unit cell. The arrow represents the direction of symmetry points along which the bandstructure is calculated. (b-i) depicts the bandstructure result obtained for each quadrant.


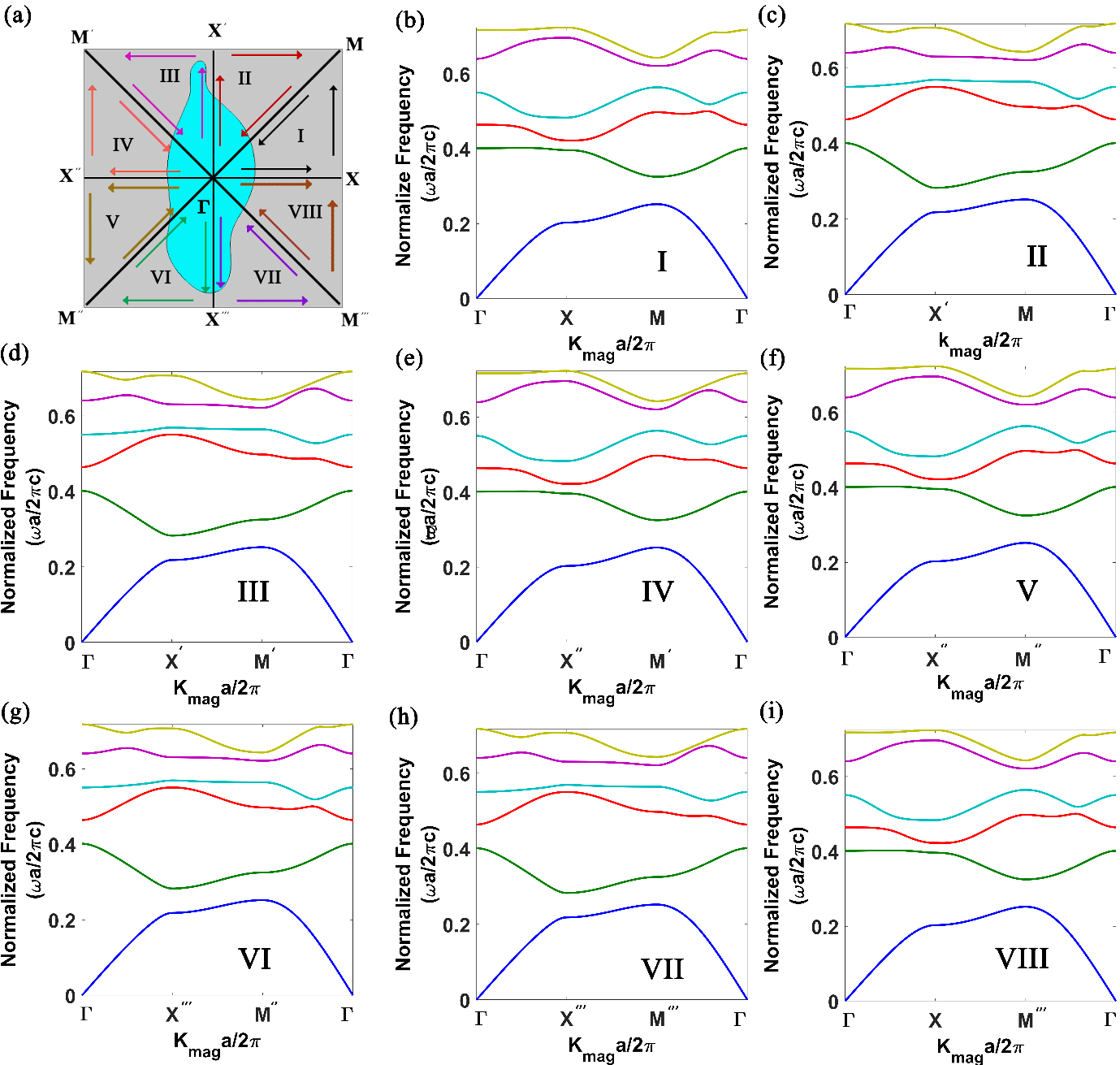


Fig. S2. Bandstructure obtained for the entire zone by considering eight different quadrants of the unit cell shown in (a). (a) N-structure square lattice unit cell. The arrow represents the direction of symmetry points along which the bandstructure is calculated. (b-i) depicts the bandstructure result obtained for each quadrant.


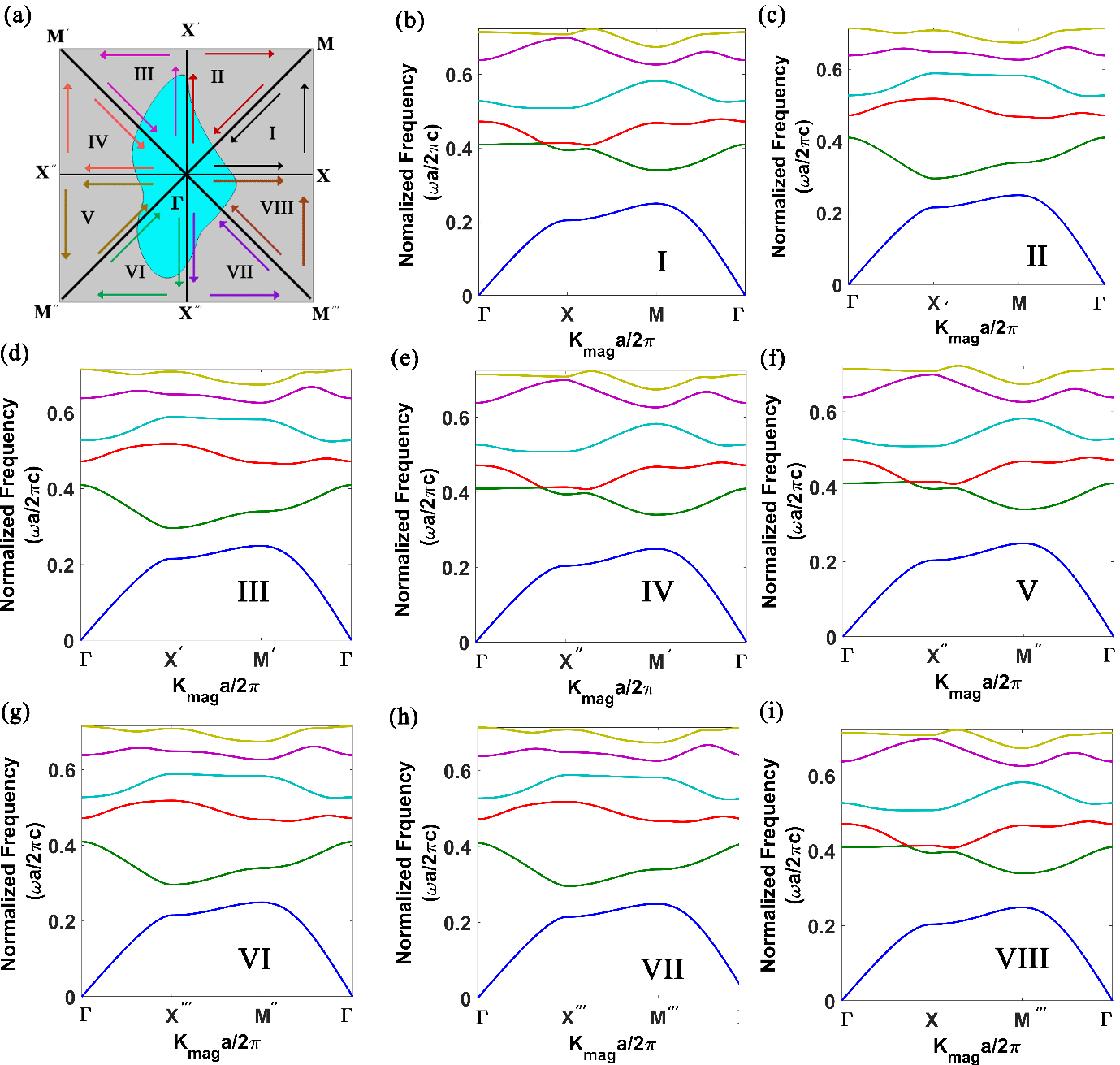


Fig. S3. Bandstructure obtained for the entire zone by considering eight different quadrants of the unit cell shown in (a). (a) W-structure square lattice unit cell. The arrow represents the direction of symmetry points along which the bandstructure is calculated. (b-i) depicts the bandstructure result obtained for each quadrant.

**Table 1: Gap to mid gap ratio percentage for triangular lattice PhCs with different geometric patterns for TM polarization.**

| Structure | Band Interval | Frequency Interval (c/*a*) | Gap Percentage (%) |
| --- | --- | --- | --- |
| C | TM1-TM2 | 0.3920-0.4222 | 7.40 |
| L | TM2-TM3 | 0.4847-0.5154 | 6.13 |
|  | TM3-TM4 | 0.6169-0.6310 | 2.26 |
|  | TM4-TM5 | 0.7169-0.7485 | 4.58 |
| N | TM2-TM3 | 0.5570-0.6018 | 7.74 |
|  | TM3-TM4 | 0.6193-0.7318 | 16.65 |
|  | TM4-TM5 | 0.7640-0.7748 | 1.43 |
|  | TM5-TM6 | 0.8357-0.8643 | 3.36 |
| W | TM2-TM3 | 0.5137-0.5223 | 1.65 |
|  | TM3-TM4 | 0.6196-0.6322 | 2.0 |

**II. Transmission loss computation for a 3-D PhC with and without dielectric losses**


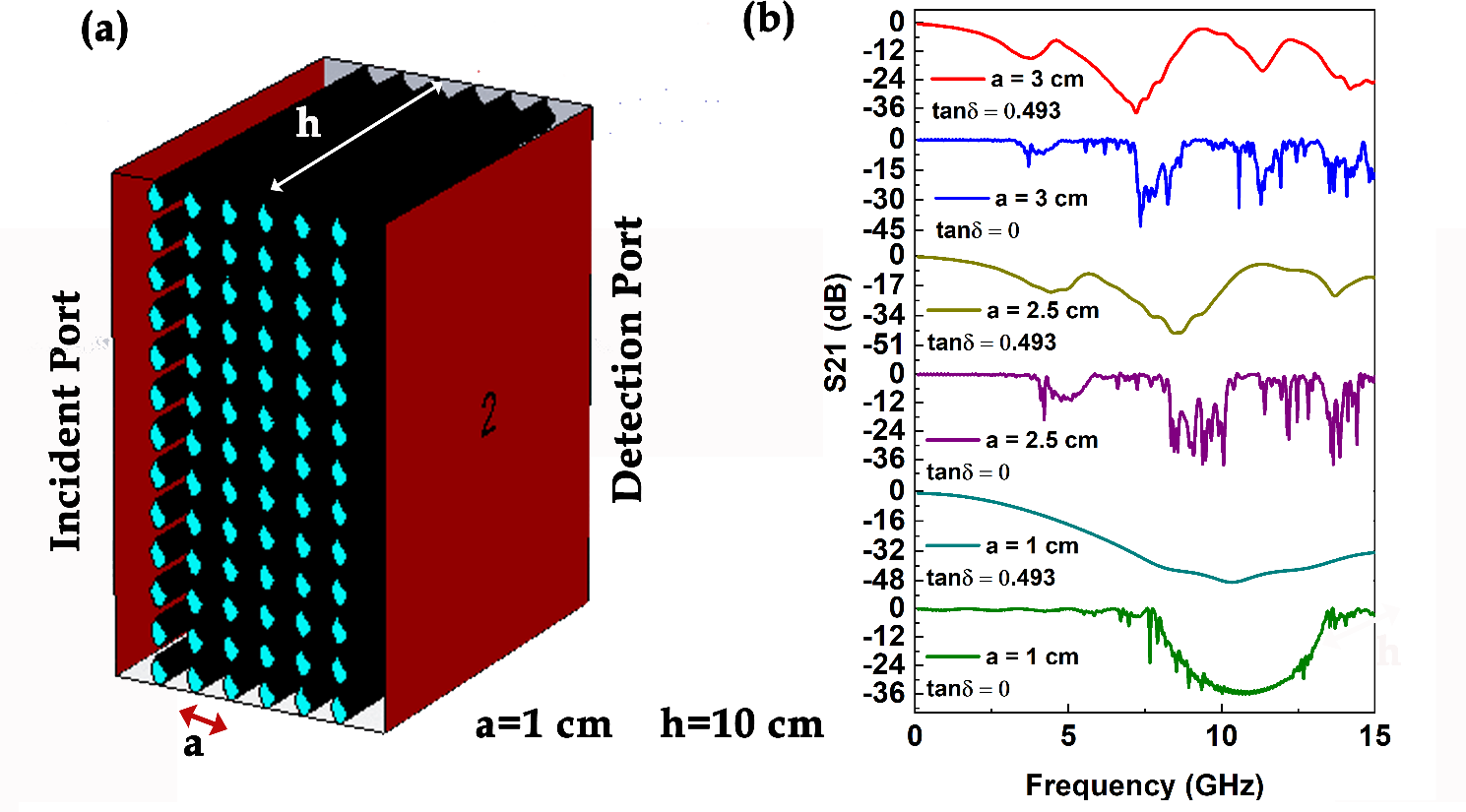


Fig. S4. Transmission loss computation of 3-D PhC with and without dielectric loss for three different cases. (a) Computational setup and (b) Transmission loss plot.

**III. Comparison of Bandstructure Results Obtained from COMSOL and MPB Solvers**


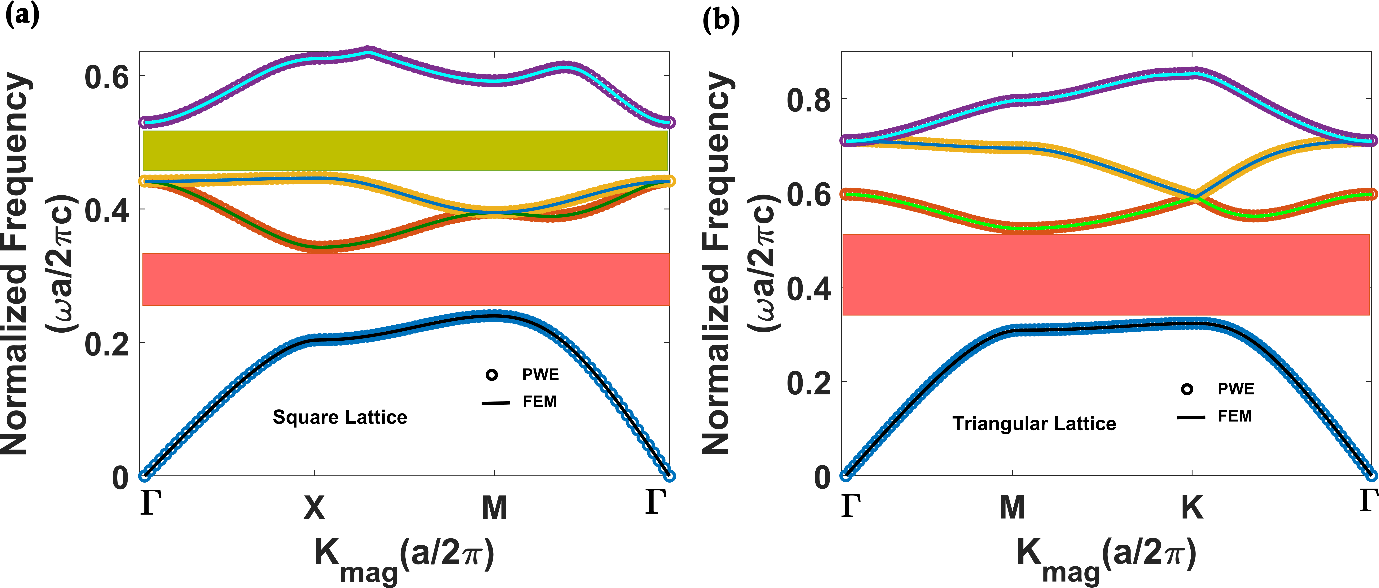


**Fig. S5.** TE Photonic Bandstructure results obtained from two different methods for circular dielectric rods (dielectric permittivity 12.96) arranged in (a) square lattice and (b) triangular lattice with the radius *r* = 0.252*a* and 0.15*a* in air background respectively. The shaded area corresponds to the PBG region.

**Table 2: Comparison of PBG results obtained from two different methods (PWE and FEM) for square and triangular lattice circular rod PhCs.**

| **Lattice** | **Mode** | **Band** | **PBG (%)** | | **Error %** |
| --- | --- | --- | --- | --- | --- |
|  |  |  | **PWE** | **FEM** |  |
| Square | TE | 1-2 | 35.23 | 35.27 | 0.116 |
|  |  | 3-4 | 16.96 | 17.04 | 0.491 |
|  | TM | - | - | - | 0 |
| Triangular | TE | 1-2 | 47.39 | 47.47 | 0.162 |
|  | TM | - | - | - | 0 |
